# Clinics Given at Ohio State Dental Society

**Published:** 1901-03-15

**Authors:** 


### CLINICS GIVEN AT OHIO STATE DENTAL SOCIETY.

### GOLD FILLING WITH SMOOTH PLUGGERS.

Dr. J. B. Beauman, of Columbus. The gold used was No. 6 foil, the leaves taken from the book and carefully laid on top of each other and pressed together by placing the empty book on top and rubbing the hand over the book, the sheets thus placed forming a No. 60 foil. This was then cut once through the middle and then strips were cut crosswise as desired. The gold was annealed, and packed with hand mallet and burnished pluggers with small points. The cavity was a large distal cavity in a superior left incisor.

The Doctor also filled a tooth that was imbedded in plaster, using the same materials and instruments. This filling he removed from the tooth and rolled it out to about a No. 60 foil and refilled another cavity with the gold thus obtained. This gold presented much the same appearance as it would on being melted and rolled out. It was an exceedingly interesting clinic.

### FILLING BROKEN-DOWN TEETH WITH THE AID OF A MATRIX.

Dr. H. M. Semans, of Columbus, adapted a simple matrix about a badly decayed first inferior molar whose lingual wall was broken entirely away. The matrix of desirable thickness is cut to gum margin and bent to fit the tooth. The ends passing a short distance beyond the remaining mesial and distal walls. A hole is punched in each end of matrix near the gum border, one end of a thread is passed through these holes, the matrix then placed in position, the thread being slipped over the buccal wall and forced well down on mesial and distal sides, after which it is tied with a surgeon's knot, the ends of the thread may then be wrapped about the matrix and tooth as much as desired, adding rigidity. Rubber dam, cotton or napkins may then be used to suit the case or desire of operator. A matrix put on in this way may be retained in the mouth a number of days with no discomfort to patient.

### GOLD AND TIN AS A FILLING MATERIAL.

Dr. J. R. Callahan, of Cincinnati, explained the use of gold and tin in the best proportions for filling. The combinations consist of (1) gold two parts, tin one; (2) gold ten parts, tin one; (3) gold and tin equal parts. Uses Abbey's non-cohesive gold No. 10, also uses tin foil No. 10. The use of these combinations is only good in four wall cavities. Uses combination of the number three for submarine work and also where there is no great strain. Combinations (1) and (3) turns dark, while combination (2) holds natural color. A sheet of gold and a sheet of tin is laid together, cut into strips and then inserted into cavity and forced into place by the use of the old-fashioned burnished hand pluggers, soft gold or tape pluggers, like Dr. B. J. Bing's. After cavity is filled protruding over border of cavity an engine burnisher slightly larger than the cavity is used to condense down the surface, running the engine very rapidly, after which filling is ground and polished to its proper finish.

### PORCELAIN INLAYS.

Dr. D. W. Clancey, of Cincinnati, removed two gold fillings on the labial surfaces of the upper central incisors. Prepared cavity in the usual way for inlays.

Custer furnace was used to bake inlays. Used the gold foil for one inlay, the platinum foil for the other. After being baked they were disked to the proper shape and inserted in cavities.

---

CONTOUR FILLINGS WITH SOFT FOIL.

Dr. G. S. Junkerman, of Cincinnati, filled a large mesio-occlusal cavity in superior left bicuspid. After preparing cavity in the usual manner, No. 4 foil was cut in half sheets and rolled lengthwise into a long roll. These were taken in foil carriers, grasping roll of gold by the middle, and rolled upon itself over the end ot foil, carrier then inserted into the cavity and forced into place by hand pressure, after which it was condensed by the use of the mallet. The Doctor worked rapidly and the filling, when completed, seemed to be well made.

---

A BANDED LOGAN CROWN.

Dr. W. H. Gensley, of Cincinnati, prepared a root in the usual manner, and made a capped band as for a Richmond crown, but without a post. An elliptical hole large enough to admit the Logan crown post was made in the cap. The capped band was then placed on the root and an orange wood post fitted into the pulp canal. A wax bite and plaster impression were then made, and the articulation transferred to an articulator. The wooden post was removed and a suitable Logan crown selected and ground to fit the articulation. Cement crown to cap and remove from plaster model and invert for soldering. Pack the joint with gold foil and fuse into it a few small pieces of gold solder.

---

THE USE OF RUBBER DAM WHEN SETTING LOGAN CROWNS.

Dr. Jas. E. Boyd, of Cincinnati, used an Ivory No.

6 clamp to apply the dam to the root so as to force the gum out of the way, thus securing a clean and dry operation.

---

### CAST ALUMINUM PLATES.

Dr. W. O. Hulick, of Cincinnati, presented a casting flask which had a detachable pouring top. His method of procedure was as follows: Impression of mouth is taken, model made and wax built up on model as for ordinary cast lower or upper plate, only it is made much thicker. It is then set up in casting flask, second half poured and separated. Instead of cutting gates at each heel, gate is cut extending along entire posterior portion of plate for uppers, making it very thick. The cast is then set aside to dry and allowed to cool. The portion of flask, through which metal is poured, is then heated and set down on top of the flask, and the metal is poured while it is quite hot, while the mold proper is cool. The claims for this method is that the plate is chilled suddenly and the large bulk of metal in the pouring gate, being kept warm, cools slowly and shrinkage takes place there, while the plate proper is already chilled. Plate is then taken from flask and this large, thick gate is cut off by means of a saw and coarse files. Plate proper is then smoothed and finished, where rubber attachment is to be made, it is burred out by the use of vulcanite burs keeping a good definite border around lingual portion. The aluminum used is that made by the Pittsburg Reduction Co., and is claimed to be nearly pure.

---

### A NEW METHOD OF MAKING DUMMIES.

Dr. J. K. Smith, of Zanesville. After abutments to which the dummies of a bridge are to be attached are made, porcelain teeth are ground into place to articulate with the opposing teeth. The porcelain teeth thus articulated are waxed together with some sort of sticky wax, and an impression of them is taken in moldine clay, a die poured, upon which No. 28 gold plate is swaged directly into lead, using no counter die. A mold in gold thus formed is either flowed solidly full with solder or backed up with gold and soldered. This is then filed to fit between two crowns already formed for bridge and the whole soldered together and thus solid gold bridge is obtained.

---

### A MODIFIED MATRIX, AND ANOTHER METHOD OF SUPPORTING A BRIDGE.

Dr. C. T. Whinnery, of Toledo, showed an ordinary matrix with the lower edge turned up, so that the jaws of the clamp or separator would hold it down to the gum firmly and prevent its dislodgment. He also showed a method of supporting small bridges by hanging them to caps cemented upon the incisal ends of the teeth. Useful where full bands can not be utilized or the inclination of the teeth interfere with adjustment.

---

### A NEW METHOD OF SWAGING METAL PLATES.

Dr. E. L. Patchin, of Cleveland, used a piece of car spring rubber as a counter die. It was given the general shape of a counter die except that it overlapped about one-half an inch all around. The metal is placed upon the die and the rubber counter die No. 1 was put upon it and it was put into a flask press and carried hard down upon the die. The plate should strike the die in the center of the palatal surface and spread from the center outward. Then take a machinist's hammer and pound down the rubber upon the die carrying the plate down over the process. It is then taken from the flask and by means of the crown shears trimmed for the rim. It is then placed upon a swage block and struck by the round end of the machinist's hammer, over which is placed a rubber crutch tip, which has in it a piece of heavy rubber. By this means the plate is forced into absolute contact with the die. The plate should be steadied with the fingers while driving it home in order to keep it from jumping from place. It is then placed in the holder again with the counter die (rubber) the same as No. 1, with the exception that it covers over the palatal surface. It is forced down tightly in press and thus held in place while the rim is pounded down with a horn mallet. Now release rubber holder and put on No. 1 again; then put on a piece of packing rubber about a half inch wide, about fourteen inches long and one-third of an inch thick. This passes around the rim of plate close up to No. 1 projection, as it projects at the heel of the plate gather the ends into the left hand and tip the rubber covered die back upon the heel of die and pound down rim.

This is particularly desirable for aluminum plates, as the surface is to be kept free from scratches and blemishes as far as possible. Mention was made of substituting finely cut up rubber or cork in the Parker Swaging Apparatus.

---

### NEW APPLIANCES.

Dr. W. A. Price, of Cleveland, presented two very ingenious appliances; the first consisted of an instrument designed for putting in gutta-percha fillings made after the principle of an electric cautery. It was suggested to the Doctor that the same principle could be utilized in the making of a wax spatula. The second consisted of an appliance to the hand-piece of a dental engine for the purpose of blowing a constant stream of warm air into the cavity while excavating. A stream of air is forced over a small incandescent lamp in this way heated at the same time light is thrown into the cavity.

---

### GOLD AND FELT FOIL FILLING.

Dr. L. T. Canfield, of Toledo. The cavity was a right first superior molar anterior proximal. Lingual and buccal walls were badly broken down, cervical border decayed to considerable depth below the gum line. A very narrow matrix was used about an eighth of an inch wide at the cervical border. No separator was used, as there was some separation. The cavity was prepared in the usual way, the buccal and lingual walls were cut away quite extensively owing to their being quite frail. The bottom of the cavity was filled with Robinson's felt foil (tin) by the use of hand pressure, being careful to cover all cervical margins. The filling was finished with Morgan & Hasting's cohesive gold, condensing with pneumatic mallet. This latter being one of Dr. Canfield's own design and the use of which he demonstrated. The filling was contoured well over the buccal and lingual borders, buckling tightly against the prominent tooth. It was then separated a trifle with the separator to allow of proper finishing. After the filling was finished and separator removed, the filling knuckled tightly against the bicuspid at its proximate occlusal surface.

